# Associations between remnant lipoprotein cholesterol and central systolic blood pressure in a Chinese community-based population: a cross-sectional study

**DOI:** 10.1186/s12944-021-01490-0

**Published:** 2021-06-26

**Authors:** Kaiyin Li, Fangfang Fan, Bo Zheng, Jia Jia, Bo Liu, Jiahui Liu, Chuyun Chen, Jing Zhou, Yan Zhang, Yong Huo

**Affiliations:** 1 Department of Cardiology, Peking University First Hospital, No. 8 Xishiku Street, Xicheng District, Beijing, 100034 China; 2 Institute of Cardiovascular Disease, Peking University First hospital, No. 8 Xishiku Street, Xicheng District, Beijing, 100034 China

**Keywords:** Lipids, Remnant lipoprotein cholesterol, Non-high-density lipoprotein cholesterol, Triglyceride, Cholesterol, Low-density lipoprotein cholesterol, Non-invasive central systolic blood pressure, Arterial stiffness

## Abstract

**Background:**

The lipid profile is reportedly related to peripheral blood pressure or pulse wave velocity. However, no studies have investigated the associations between lipid parameters, especially remnant lipoprotein cholesterol (RLP-C), and central systolic blood pressure (cSBP).

**Methods:**

This study used baseline data of a community-based cohort in Beijing, China. Participants who had been treated with anti-hypertensive or lipid-lowering agents were excluded. RLP-C is equal to total cholesterol (TC) minus the sum of low-density lipoprotein cholesterol (LDL-C) and high-density lipoprotein cholesterol (HDL-C). An Omron HEM-9000AI device was used to measure non-invasive cSBP. The associations between blood lipid profile and non-invasive cSBP were evaluated using multivariable regression models.

**Results:**

The 5173 included participants were 55.0 ± 8.5 years old; 35.7% (1845) of participants were men. Increased cSBP was significantly associated with increased TC, LDL-C, non-high-density lipoprotein cholesterol (non-HDL-C), triglyceride (TG), and RLP-C but with decreased HDL-C, even after adjusting for possible covariates. When simultaneously entering individual pairs of RLP-C and other blood lipid parameters into the multivariable regression model, RLP-C remained significantly associated with cSBP, even after adjusting for other lipids. Compared with participants who had RLP-C levels in the first quartile (Q1), cSBP for those with RLP-C in Q4 was increased to 4.57 (95% confidence interval [CI]: 3.08–6.06) mmHg after adjusting for LDL-C, 4.50 (95%CI: 2.98–6.02) mmHg after adjusting for TC, 3.91 (95%CI: 1.92–5.89) mmHg after adjusting for TG, 5.15 (95%CI: 3.67–6.63) mmHg after adjusting for HDL-C, and 4.10 (95%CI: 2.36–5.84) mmHg after adjusting for non-HDL-C.

**Conclusions:**

Increased blood RLP-C level was significantly associated with higher cSBP in a Chinese population, independently of other lipids, which indicates its importance in individual cardiovascular risk assessment.

## Background

Hypertension accounts for the largest proportion of the global disease burden and mortality in recent years [1]. In 2019, elevated systolic blood pressure (SBP) led to approximately 10.8 million deaths and the highest percentage of disability-adjusted life-years [2]. Hypertension is currently defined according to the peripheral blood pressure; however, pulse wave analysis (PWA) has enabled reproducible and non-invasive measurement of central hemodynamics. Numerous clinical studies have indicated that non-invasive central systolic blood pressure (cSBP) measurement may improve risk stratification for cardiovascular diseases owing to its stronger association with hypertension-mediated organ damage [3–7].

Previous studies have identified an association between blood lipids and blood pressure, although most such studies have focused on the relationships between hypercholesterolemia or hypertriglyceridemia and peripheral blood pressure [8–11]. Additionally, patients with increased remnant lipoprotein cholesterol (RLP-C) levels are more likely to develop incident cardiovascular diseases [12–14]. A 10-year longitudinal study in Japan showed associations between higher plasma RLP-C and incident hypertension [15]. To the best of our knowledge, the relationship between RLP-C and central blood pressure remains unknown. Hence, this retrospective study was conducted to investigate the associations between non-invasive cSBP and lipid profile levels, especially RLP-C.

## Methods

### Study design

This retrospective study used baseline data of a Beijing community-based cohort in China [16]. Among the initial 9540 participants recruited via phone calls or recruitment posters between December 2011 and April 2012, 8062 participants (84.5%) had effective data of cSBP and RLP-C at baseline. Those participants treated with anti-hypertensive agents (*n* = 2630) and/or lipid-lowering agents (*n* = 952) were excluded owing to therapeutic effects on cSBP and blood lipids of these drugs. Finally, 5173 eligible participants were included in the analysis.

### Data collection

Trained research staff collected data by standards, including information on lifestyle, history of diseases and medications, and physical examination. Smoking status, drinking status, hypertension, and diabetes mellitus (DM) have been defined previously [17]. Calculations of estimated glomerular filtration rate (eGFR) and body mass index (BMI) were according to those in a previous publication [18].

After an overnight fast, blood samples were obtained from each participant via forearm venipuncture. Serum glucose, lipid, and creatinine levels were tested using previously published methods [19]. Non-high-density lipoprotein cholesterol (non-HDL-C) was calculated as subtraction of the high-density lipoprotein cholesterol (HDL-C) from the total cholesterol (TC), while the RLP-C was defined as TC minus the sum of low-density lipoprotein cholesterol (LDL-C) and HDL-C.

Before central blood pressure measurement, all participants were required to sit quietly for 5 min. Non-invasive cSBP was measured using the validated HEM-9000AI device (Omron Healthcare, Kyoto, Japan) [20–24]. Each participant underwent simultaneous measurement of left radial applanation tonometry and right brachial cuff oscillometric pressure. Radial waveforms were analyzed to obtain the peripheral systolic pressure peaks, and were then calibrated using the measured brachial blood pressure. A linear regression model with the late peak of the peripheral systolic pressure was used to calculate the non-invasive cSBP. This measurement was not repeated due to the tight research schedule.

### Statistical analysis

Mean ± standard deviation was used to describe continuous variables with a normal distribution, and differences were determined with one-way analysis of variance. Median (interquartile range) was used to describe non-normally distributed variables, and differences were determined using the Kruskal-Wallis rank test. Number (%) was used to describe dichotomous variables, and differences were determined using the chi-square test.

Relationships between lipid parameters and cSBP level were examined with generalized additive models using a spline smoothing function. Associations between cSBP and different blood lipid parameters (as continuous variables and quartiles [Q]) were investigated using univariable and multivariable regression models. Age, sex, BMI, eGFR, smoking and drinking status, DM, antidiabetic drug use, myocardial infarction and stroke history were adjusted in multivariable regression analyses. Furthermore, independent associations of RLP-C with cSBP, adjusted for other lipid parameters, were determined by simultaneously entering the pairs of RLP-C and other lipid parameters into the multivariable regression models one at a time. With regard to possible collinearity, the variance inflation factor (VIF) was calculated for the included variables in each multivariable regression model. Empower(R) (www.empowerstats.com, X&Y Solutions, Inc., Boston, MA, USA) and R (http://www.R-project.org) were used for data analyses, and two-sided *P* values < 0.05 were regarded as statistically significant.

## Results

### Baseline characteristics of enrolled participants

Table 1 summarizes the characteristics of enrolled participants according to quartiles of RLP-C. The 5173 included participants were 55.0 ± 8.5 years old, and 35.7% (1845) were men. Participants with a higher RLP-C level had increased levels of TC, triglyceride (TG), LDL-C, and non-HDL-C, as well as cSBP (*P* < 0.001). Despite no intergroup difference in the percentage of myocardial infarction or stroke history, participants with a higher RLP-C level tended to be older, male, more likely to smoke and drink, and to have higher BMI, worse renal function, a higher ratio of DM, hypertension (*P* < 0.001) and antidiabetic medication use (*P* = 0.013).

**Table 1:** Baseline characteristics of all participants by RLP-C quartile

Variables	Total	RLP-C, mmol/L				*P* Value
		Q1 (< 0.39)	Q2 (0.39–< 0.54)	Q3 (0.54–< 0.73)	Q4 (≥0.73)	
n	5173	1269	1283	1318	1303	
Age, y	55.0 ± 8.5	54.2 ± 8.8	55.3 ± 9.1	55.5 ± 8.2	55.0 ± 7.9	< 0.001
Male, n (%)	1845 (35.7%)	365 (28.8%)	444 (34.6%)	476 (36.1%)	560 (43.0%)	< 0.001
BMI, kg/m^2^	25.5 ± 3.3	24.0 ± 3.0	25.3 ± 3.2	26.1 ± 3.2	26.7 ± 3.1	< 0.001
eGFR, mL/min·1.73m^2^	96.8 ± 12.0	98.7 ± 11.4	96.9 ± 11.9	96.1 ± 11.9	95.4 ± 12.5	< 0.001
cSBP, mmHg	129.7 ± 17.3	126.1 ± 16.8	129.0 ± 17.3	130.2 ± 16.8	133.2 ± 17.4	< 0.001
TC, mmol/L	5.4 ± 1.0	4.8 ± 0.8	5.1 ± 0.8	5.5 ± 0.9	6.0 ± 1.1	< 0.001
TG, mmol/L	1.2 (0.9–1.8)	0.8 (0.6–0.9)	1.1 (0.9–1.3)	1.4 (1.2–1.7)	2.3 (1.8–3.1)	< 0.001
HDL-C, mmol/L	1.5 ± 0.4	1.8 ± 0.4	1.5 ± 0.3	1.4 ± 0.3	1.2 ± 0.3	< 0.001
LDL-C, mmol/L	3.3 ± 0.8	2.7 ± 0.6	3.2 ± 0.6	3.5 ± 0.7	3.7 ± 1.0	< 0.001
RLP-C, mmol/L	0.5 (0.4–0.7)	0.3 (0.2–0.4)	0.5 (0.4–0.5)	0.6 (0.6–0.7)	0.9 (0.8–1.1)	< 0.001
Non-HDL-C, mmol/L	3.9 ± 1.0	3.0 ± 0.6	3.6 ± 0.6	4.1 ± 0.7	4.8 ± 1.0	< 0.001
Smoking, n (%)	1038 (20.1%)	163 (12.8%)	219 (17.1%)	275 (20.9%)	381 (29.2%)	< 0.001
Drinking, n (%)	1255 (24.3%)	250 (19.7%)	299 (23.3%)	317 (24.1%)	389 (29.9%)	< 0.001
Prevalence of cardiovascular disease, n (%)						
Diabetes mellitus	953 (18.4%)	159 (12.5%)	208 (16.2%)	253 (19.2%)	333 (25.6%)	< 0.001
Self-reported Myocardial Infarction	21 (0.4%)	0 (0.0%)	7 (0.6%)	7(0.5%)	7 (0.5%)	0.077
Self-reported Stroke history	97 (1.9%)	21 (1.7%)	30 (2.3%)	28 (2.1%)	18 (1.4%)	0.261
Hypertension	1342 (25.9%)	237 (18.7%)	311 (24.2%)	355 (26.9%)	439 (33.7%)	< 0.001
Antidiabetic medication use	325 (6.3%)	60 (4.7%)	76 (5.9%)	88 (6.7%)	101 (7.8%)	0.013

Data are presented as mean ± standard deviation for normally distributed continuous variables, median (interquartile range) for non-normally distributed continuous variables, and number (percentage) for dichotomous variables

*Abbreviations*: *BMI* body mass index, *eGFR* estimated glomerular filtration rate, *cSBP* central systolic blood pressure, *TC* total cholesterol, *TG* triglyceride, *HDL-C* high–density lipoprotein cholesterol, *LDL-C* low-density lipoprotein cholesterol, *RLP-C* remnant lipoprotein cholesterol, *non-HDL-C* non-high-density lipoprotein cholesterol

### Associations of RLP-C and other lipid profiles with cSBP, considered individually

**Fig. 1:**
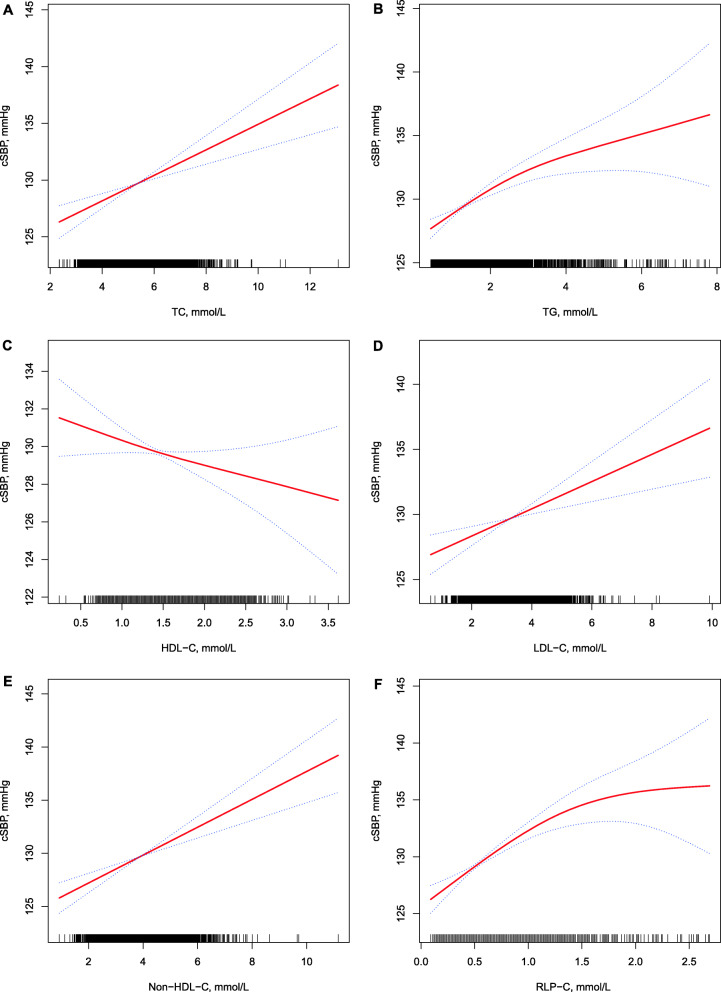
Smoothing curve of cSBP by lipid parameter. Adjusted for sex, age, body mass index, estimated glomerular filtration rate, smoking, alcohol use, diabetes mellitus, antidiabetic drug use, myocardial infarction and stroke. **A**: Association between TC and cSBP. **B**: Association between TG and cSBP. **C**: Association between HDL-C and cSBP. **D**: Association between LDL-C and cSBP. **E**: Association between non-HDL-C and cSBP. **F**: Association between RLP-C and cSBP. cSBP: central systolic blood pressure; TC: total cholesterol; TG: triglyceride; HDL-C: high-density lipoprotein cholesterol; LDL-C: low-density lipoprotein cholesterol; non-HDL-C: non-high-density lipoprotein cholesterol; RLP-C: remnant lipoprotein cholesterol

The smoothing curves of cSBP by lipid parameter after adjusting for possible covariates are shown in Fig. 1. The smoothing curves showed a positive linear association between most lipid parameters and cSBP, except that a negative linear association with the HDL-C level and a non-linear trend with RLP-C level were observed. Associations between the blood lipid profile and cSBP are summarized in Table 2. In the univariable analyses, cSBP showed a graded positive relationship with RLP-C, TC, TG, LDL-C, and non-HDL-C levels, but a graded negative relationship with the HDL-C level (*P* < 0.001). In the adjusted multivariable analyses, higher cSBP was significantly associated with increases in TC, TG, LDL-C, RLP-C, and non-HDL-C levels but decreased HDL-C level.

**Table 2:** Multivariable adjusted change in cSBP by quartiles of blood lipids, considered individually

Variable	Change in cSBP, mmHg (95% CI)	*P* value
TC, mmol/L		
Continuous, per 1 mmol/L	1.20 (0.73–1.67)	< 0.001
Quartiles		
Q1: < 4.70	Ref.	
Q2: 4.70–< 5.28	0.72 (−0.57–2.00)	0.274
Q3: 5.28–< 5.95	1.99 (0.71–3.28)	0.002
Q4: ≥5.95	2.58 (1.28–3.88)	< 0.001
TG, mmol/L		
Continuous, per 1 mmol/L	0.84 (0.49–1.19)	< 0.001
Quartiles		
Q1: < 0.87	Ref.	
Q2: 0.87–< 1.24	1.51 (0.23–2.80)	0.021
Q3: 1.24–< 1.79	2.30 (0.98–3.61)	< 0.001
Q4: ≥1.79	4.22 (2.87–5.57)	< 0.001
HDL-C, mmol/L		
Continuous, per 1 mmol/L	−1.43 (−2.76– − 0.10)	0.035
Quartiles		
Q1: < 1.19	Ref.	
Q2: 1.19–< 1.42	−0.74 (−2.05–0.57)	0.268
Q3: 1.42–< 1.68	−1.00 (−2.36–0.36)	0.151
Q4: ≥1.68	−1.60 (−3.03– − 0.17)	0.028
LDL-C, mmol/L		
Continuous, per 1 mmol/L	1.00 (0.44–1.56)	< 0.001
Quartiles		
Q1: < 2.73	Ref.	
Q2: 2.73–< 3.24	1.40 (0.12–2.68)	0.032
Q3: 3.24–< 3.78	1.35 (0.06–2.64)	0.041
Q4: ≥3.78	2.63 (1.34–3.93)	< 0.001
Non-HDL-C, mmol/L		
Continuous, per 1 mmol/L	1.38 (0.91–1.85)	< 0.001
Quartiles		
Q1: < 3.22	Ref.	
Q2: 3.22–< 3.83	2.33 (1.05–3.60)	< 0.001
Q3: 3.83–< 4.49	2.14 (0.85–3.43)	0.001
Q4: ≥4.49	3.88 (2.57–5.18)	< 0.001
RLP-C, mmol/L		
Continuous, per 1 mmol/L	3.94 (2.82–5.07)	< 0.001
Quartiles		
Q1: < 0.39	Ref.	
Q2: 0.39–< 0.54	1.62 (0.33–2.91)	0.014
Q3: 0.54–< 0.73	2.19 (0.88–3.50)	0.001
Q4: ≥0.73	4.88 (3.53–6.23)	< 0.001

Model adjusted for age, sex, body mass index, estimated glomerular filtration rate, smoking status, drinking status, diabetes mellitus, antidiabetic drug use, history of myocardial infarction and history of stroke

*Abbreviations*: *TC* total cholesterol, *TG* triglyceride, *HDL-C* high-density lipoprotein cholesterol, *LDL-C* low-density lipoprotein cholesterol, *non-HDL-C* non-high-density lipoprotein cholesterol, *RLP-C* remnant lipoprotein cholesterol, *cSBP* central systolic blood pressure

### Associations of RLP-C and other lipid profiles with cSBP, considered simultaneously

The VIF calculated for the included variables in each multivariable regression model did not show any collinearity (Supplementary materials). Table 3 shows the relationship of RLP-C level and other blood lipid parameters with non-invasive cSBP, with simultaneous entry of RLP-C and other lipid in pairs into the multivariable regression models, after adjusting for covariates. Compared with participants who had RLP-C in Q1, the cSBP for those with RLP-C in Q4 was increased to 4.57 (95% confidence interval [CI]: 3.08–6.06) mmHg after adjusting for LDL-C, 4.50 (95%CI: 2.98–6.02) mmHg after adjusting for TC, 3.91 (95%CI: 1.92–5.89) mmHg after adjusting for TG, 5.15 (95%CI: 3.67–6.63) mmHg after adjusting for HDL-C, and 4.10 (95%CI: 2.36–5.84) mmHg after adjusting for non-HDL-C. The other lipid parameters were not significantly associated with cSBP after adjusting for RLP-C.

**Table 3:** Multivariable adjusted change in cSBP by quartiles of RLP-C and other lipid parameters, considered simultaneously

Models	Change in cSBP, mmHg (95% CI)	*P* value		Change in cSBP, mmHg (95% CI)	*P* value
Model I^†^					
RLP-C, mmol/L			LDL-C, mmol/L		
Q1: < 0.39	Ref.		Q1: < 2.73	Ref.	
Q2: 0.39–< 0.54	1.43 (0.10–2.77)	0.035	Q2: 2.73–< 3.24	0.97 (−0.33–2.27)	0.143
Q3: 0.54–< 0.73	1.89 (0.48–3.31)	0.009	Q3: 3.24–< 3.78	0.43 (−0.92–1.79)	0.529
Q4: ≥0.73	4.57 (3.08–6.06)	< 0.001	Q4: ≥3.78	0.93 (−0.50–2.37)	0.202
Model II^†^					
RLP-C, mmol/L			TC, mmol/L		
Q1: < 0.39	Ref.		Q1: < 4.70	Ref.	
Q2: 0.39–< 0.54	1.47 (0.15–2.78)	0.029	Q2: 4.70–< 5.28	0.14 (−1.16–1.44)	0.832
Q3: 0.54–< 0.73	1.90 (0.51–3.29)	0.007	Q3: 5.28–< 5.95	0.92 (−0.42–2.26)	0.178
Q4: ≥0.73	4.50 (2.98–6.02)	< 0.001	Q4: ≥5.95	0.66 (−0.80–2.12)	0.374
Model III^†^					
RLP-C, mmol/L			TG, mmol/L		
Q1: < 0.39	Ref.		Q1: < 0.87	Ref.	
Q2: 0.39–< 0.54	1.22 (−0.17–2.62)	0.087	Q2: 0.87–< 1.24	0.96 (−0.41–2.32)	0.170
Q3: 0.54–< 0.73	1.53 (−0.07–3.13)	0.061	Q3: 1.24–< 1.79	1.02 (−0.57–2.61)	0.209
Q4: ≥0.73	3.91 (1.92–5.89)	< 0.001	Q4: ≥1.79	1.47 (−0.50–3.43)	0.144
Model IV^†^					
RLP-C, mmol/L			HDL-C, mmol/L		
Q1: < 0.39	Ref.		Q1: < 1.19	Ref.	
Q2: 0.39–< 0.54	1.72 (0.40–3.03)	0.010	Q2: 1.19–< 1.42	0.09 (−1.24–1.42)	0.895
Q3: 0.54–< 0.73	2.36 (0.99–3.73)	< 0.001	Q3: 1.42–< 1.68	0.52 (−0.90–1.94)	0.474
Q4: ≥0.73	5.15 (3.67–6.63)	< 0.001	Q4: ≥1.68	0.59 (−0.97–2.15)	0.457
Model V^†^					
RLP-C, mmol/L			Non-HDL-C, mmol/L		
Q1: < 0.39	Ref.		Q1: < 3.22	Ref.	
Q2: 0.39–< 0.54	1.15 (−0.23–2.53)	0.104	Q2: 3.22–< 3.83	1.67 (0.31–3.03)	0.016
Q3: 0.54–< 0.73	1.54 (−0.00–3.08)	0.050	Q3: 3.83–< 4.49	0.79 (−0.70–2.28)	0.300
Q4: ≥0.73	4.10 (2.36–5.84)	< 0.001	Q4: ≥4.49	1.52 (−0.16–3.20)	0.077

^†^RLP-C and other blood lipid parameters were simultaneously entered into the multivariable regression model

Model adjusted for age, sex, body mass index, estimated glomerular filtration rate, smoking status, drinking status, diabetes mellitus, antidiabetic drug use, history of myocardial infarction and history of stroke

*Abbreviations*: *cSBP* central systolic blood pressure, *RLP-C* remnant lipoprotein cholesterol, *LDL-C* low-density lipoprotein cholesterol, *TC* total cholesterol, *TG* triglyceride, *HDL-C* high-density lipoprotein cholesterol, *non-HDL-C* non-high-density lipoprotein cholesterol

## Discussion

The present study shows associations between cSBP and blood lipid profiles, especially RLP-C, in a community-dwelling Chinese population. In further analyses by simultaneously entering RLP-C level and other lipid parameters in pairs, the RLP-C level showed a stronger association with non-invasive cSBP.

Although different physiological indices, blood pressure and lipid profiles have overlapping processes owing to common cardiovascular risk factors and complications. Both hypertriglyceridemia and hypercholesterolemia are associated with higher peripheral blood pressure, as reported in previous cross-sectional studies [8–11]. A cohort study further showed that adolescents with a higher TG to HDL-C ratio had a higher risk of developing adult hypertension in a 20-year follow-up [25]; similar results were also observed in another prospective cohort study among pregnant women [26]. Mechanisms such as endothelial cell dysfunction, neuroendocrine system activation, increased salt sensitivity and reduced sodium clearance by nephrons, and higher L-type calcium channel activities in the smooth myofibrils contributed to the relationship [9, 27–33]. However, previous studies focused on peripheral blood pressure, although central blood pressure has been more strongly associated with cardiovascular outcome and hypertension-mediated organ damage [3–7].

Arterial stiffness can be evaluated non-invasively using two-dimensional imaging techniques, pulse wave velocity (PWV), cardio-ankle vascular index (CAVI), and PWA, although carotid-femoral PWV is currently more reliable [34–36]. Associations between TG levels and arterial stiffness have been reported, using all measurements of the CAVI [37], brachial-ankle PWV [38], and carotid-femoral PWV [39]. Regarding cholesterol, aortic stiffness has been positively associated with TC, LDL-C, and non-HDL-C levels, and negatively associated with HDL-C level [8, 40–42]. Moreover, large artery stiffness and blood pressure control are improved with statin therapy in patients with hypertension [43], independently of changes in the LDL-C level [44]. The findings of the present cross-sectional study suggested that the blood lipid profile is associated with non-invasive cSBP, and this association might be explained by vascular impairment. Compared with PWV, central blood pressure is more than a tool to measure aortic stiffness; it is associated with a more complex set of determinants, including the physical properties of systemic arteries, PWV, the travel time of a forward wave and its reflection, and the distance to the major reflecting site of a pulse wave [45]. Relationships of these determinants and lipids have been discussed previously. Another cross-sectional study used PWA to show associations between the LDL-C level and wave reflection [46]. Hence, cSBP and PWV are both associated with the lipid profile owing to their shared determinant of aortic stiffness, whereas other determinants of cSBP may enhance this association.

Notably, RLP-C was more strongly associated with cSBP than the other lipid parameters analyzed in the present study, possibly because of its physiological characteristics. RLP-C is defined as the cholesterol content in TG-rich lipoproteins (TRLs), namely intermediate-density lipoprotein (IDL), very low-density lipoprotein (VLDL), and chylomicron remnants [47–49]. As TG contents are gradually degraded by lipoprotein lipase in the bloodstream, the cholesterol in RLP-C could also be involved in atherosclerosis plaque formation and subsequent cardiovascular events, and it has been recognized as more atherogenic and proinflammatory than the cholesterol in LDL-C [48–52]. A recent study indicated associations of plasma TGs and relatively small-sized LDL particles with the carotid-femoral PWV, despite no significant associations between VLDL/large IDL subclasses (the main carriers of TG in the blood) and the carotid-femoral PWV [53]. This raises the possibility that the TG content in TRLs may indirectly influence this process, considering greater small dense LDL generation in the presence of high VLDL-TG levels [54]. Another study conducted in Japan also linked higher serum RLP-C levels with increased intima-media thickness-defined carotid atherosclerosis and CAVI-defined aortic atherosclerosis [55]. However, an updated analysis of the Copenhagen General Population Study suggests that the cholesterol content in VLDL, but not the TG content, is the main contributor to atherosclerotic disease [56].

Some studies have focused on the influences of lipid-lowering agents on blood pressure. Statins could substantially reduce peripheral blood pressure (1 to 3 mmHg) [57, 58], improve arterial stiffness, and reduce central aortic pressure [59], despite the negative effects of atorvastatin on central hemodynamic parameters identified in the Conduit Artery Function Evaluation-Lipid-Lowering Arm study [60]. Considering the high comorbidity of hypertension and dyslipidemia possibly mediated by adiposity [61], the exact relationship between blood pressure and lipids should be investigated. A recent study showed that statin therapy could enhance blood pressure control in patients with hypertension, independently of the intensity of antihypertensive agents [62]. A mediation analysis also showed that LDL-C reduction after statin treatment is associated with better central blood pressure control [63]. The exact action of lipid-lowing agents on central blood pressure remains unknown, but clinicians should consider performing comprehensive measurement of central blood pressure and lipids profile in individuals with hypertension or dyslipidemia. To uncover the natural relationship of RLP-C with cSBP, participants taking lipid-lowing agents or antihypertensive medications were excluded in this study. Considering that these treated participants may have higher values of lipids or cSBP, analyses after including them were also performed, and the main findings were not substantially changed.

### Study strengths and limitations

This study demonstrated the association between RLP-C and cSBP for the first time, which strengthens the association between blood lipid levels and vascular health from the viewpoint of cSBP. Among the analyzed lipid parameters, RLP-C showed the strongest association with cSBP, offering a novel research direction.

This study also has several limitations. First, central diastolic pressure and pulse pressure were not discussed because the Omron devices used in this study do not measure central diastolic pressure. Second, the enrolled participants were from one site; thus, generalizability of the findings to other populations is unclear. Third, other measures of central arterial stiffness, such as carotid-femoral PWV, were not available in this study. Fourth, despite finding no collinearity in the VIF calculations, the effects of RLP-C on cSBP might not be independent of other lipids because RLP-C has a linear relationship with other lipid fractions. Thus, routine lipid fraction tests and RLP-C assessment should be conducted simultaneously in both scientific research and clinical practice. Last, causality between RLP-C and cSBP cannot be judged because of the characteristics of a cross-sectional study; additional studies are warranted to determine whether a causal relationship exists between dyslipidemia and increased non-invasive cSBP or whether these are components of a single disease or pathological state, like metabolic syndrome.

## Conclusions

Increased blood RLP-C level was significantly associated with higher cSBP in a Chinese community-based population, independently of other lipid levels. These findings emphasize the importance of RLP-C in comprehensive individual cardiovascular risk assessment. Further studies should focus on determining causality and potential mechanisms in this relationship.

## Data Availability

Data are available with reasonable request.

## Abbreviations

cSBP: Central systolic blood pressure
TRLs: Triglyceride-rich lipoproteins
TC: Total cholesterol
BMI: Body mass index
TG: Triglyceride
PWV: Pulse wave velocity
CAVI: Cardio-ankle vascular index
eGFR: Estimated glomerular filtration rate
IDL: Intermediate-density lipoprotein
VLDL: Very low-density lipoprotein
HDL-C: High-density lipoprotein cholesterol
DM: Diabetes mellitus
PWA: Pulse wave analysis
LDL-C: Low-density lipoprotein cholesterol
RLP-C: Remnant lipoprotein cholesterol
non-HDL-C: non-high-density lipoprotein cholesterol
LDL: Low-density lipoprotein
CI: confidence interval
